# Supplementary material for: The nexus between environmental concern and future childbearing aspirations among university students in Bangladesh
Source: PLoS One. 2025 Jun 4;20(6):e0325369. doi: 10.1371/journal.pone.0325369 (PMC12136327; doi:10.1371/journal.pone.0325369)
Supplement: S1 Appendix — The questionnaire was used for data collection. (DOC) [file pone.0325369.s001.doc]

**S1 Appendix: Questionnaire**

**Section I. Socio-demographic information**

Read the following questions carefully and choose any **one** option

1. What is your gender?

- Male
- Female
- Others

2. What is your religion?

- Islam
- Hindu
- Buddha
- Christian

3. Have you completed any environment-related academic course at the University?

- Yes
- No

4. Which faculty are you currently studying?

- Life Science
- Applied Science & Technology
- Management & Business Administration
- Physical Science
- Social Science

5. What is your last CGPA?

Ans: ________________________________________

6. Does your home locality vulnerable to climate change or extreme weather events?

- Yes
- No

7. If yes, what type of climate change or extreme weather events do you face? (e.g., flood, cyclone, storm)

Ans: ________________________________________

**Section II Childbearing aspirations in future**

**Read the following questions carefully and choose any one option**

8. Do you intend to have a child in the future?

- Yes (go to question 9,10)
- No (go to question 11)

9. If yes, how many children do you want in the future?

Ans: _____________________________________

10. If yes, why do you want the number of children in future?

- Children are the greatest personal achievement.
- I like children
- My parents want grandchildren, so I want kids.
- To experience parenthood.
- Other (please include) ___________________________________

11. If no, why don't you want to have children?

- I am concerned about environmental issues.
- I am worried about population growth.
- I don’t like children.
- Having children will ruin my focus on my career.
- Other (please include) __________________________________

**Section III Item regarding the impact of environmental concerns on future childbearing aspiration**

You know that the Earth's temperature is constantly changing. This results in loss of biodiversity due to extreme weather events and climate change such as floods, erratic rainfall, cyclones, extreme temperatures, droughts etc. People are suffering from food insecurity. Considering environmental issues, many couples think they will not have children in the future. They think that being childless is a better environmental strategy than recycling, driving energy-efficient cars or using compact fluorescent lights. Research also estimates that raising a child can add 9,441 tons of carbon dioxide. Individuals think that if they have children in the future, their children will also suffer from this problem. These will harm their health.

Read the following questions carefully and choose any **one** option.

12. Do you think that a harmful environment can endanger child health?

- Yes
- No

13. Do you think that childlessness has a beneficial effect on the environment?

- Yes
- No

14. Do you think that having more children in the future will increase the environmental issues?

- Yes
- No

15. Do you think that going childless is a better way to help the environment than recycling?

- Yes
- No

16. Do you think that pollution will make a baby unhealthy?

- Yes
- No

17. Do you think that childfree lifestyle can reduce the effects of climate change?

- Yes
- No

18. Do you think that people should consider having fewer children on the basis of environmental issues?

- Yes
- No

19. Do you think that having fewer children in the future is eco-friendly?

- Yes
- No
