# Supplementary material for: The nexus between environmental concern and future childbearing aspirations among university students in Bangladesh
Source: PLoS One. 2025 Jun 4;20(6):e0325369. doi: 10.1371/journal.pone.0325369 (PMC12136327; doi:10.1371/journal.pone.0325369)
Supplement: Attachment — Submitted filename: Reviewers comments Plos One.docx [file pone.0325369.s003.docx]

**Comments to the Author**

Reviewer #1: This paper presents interesting primary research that explores the nexus between environmental concerns and future childbearing aspirations among university students in Bangladesh. The author seeks to address an appropriate gap in the literature on this topic given this question has been underexplored in low- and middle-income settings, including Bangladesh.

1. Referencing and assumptions: several claims are made in the text without an adequate source for the information. For example:

Line 47: Despite there being a reference for this, the author should clarify the location of Davis et al.’s study to avoid confusion that this statement is generalisable to all women.

**Response:** Thank you for the comment. The confusion has been cleared.

Line 56: The statement “according to research, having children will damage the ecosystem” needs to be clearer on what research the author is referring to and this should be listed as a citation.

**Response:** The statement has been corrected and added reference.

Line 96: The statement that university students in Bangladesh “are more aware of family planning and concerned about the environment” needs to be backed up by a citation.

**Response:** Citations have been added and also added justification.

Line 143/44: The authors appear to advocate for a viewpoint which risks introducing personal bias. The authors should clarify why this perspective is being promoted and ensure framing of items do not reflect bias.

**Response:** The confusion has been cleared.

Line 584: The statement “women in the country may exhibit lower levels of ambition” is not supported by a source. It is unclear what ‘ambition’ means in this context, so further clarification is necessary.

**Response:** The statement has been removed as it is not supported in the manuscript.

I recommend that the authors review their manuscript to ensure that all assertions are appropriately supported with references and any subjective viewpoints should be clearly distinguished from evidence-based conclusions.


2. Structure and readability: while the use of sub-headings contributes to the logical flow of the paper, the structure within sections – particularly the introduction and literature review – could be improved. For example, lines 121-139 could be organised by dividing studies into those that found environmental concerns led to lower desires to have children vs. those that found the opposite conclusion. Language improvements are also needed. Typos, grammar issues, and repetition currently affect readability of the study. I have listed a few examples of each below but not an extensive list for the purposes of brevity. I would encourage the authors to thoroughly review the paper, and collaboration with a writing coach or copyeditor may be beneficial if possible.

**Response:** Thank you for your suggestion. The sub-sections have been added in the literature section.

Line 39: Arnocky spelt incorrectly.

**Response:** Corrected

Line 58-60: Sentence structures are unclear.

**Response:** Corrected

Line 207: Typo for ‘participating’

**Response:** Corrected

Lines 234/235: Repeated sentence.

**Response:** Corrected

3. Definitions and terminology: Key terms should be clearly defined and used consistently. This is particularly important when referring to childbearing aspirations/objectives/desires/intentions/goals which are all used throughout the manuscript, are undefined, and may imply very different meanings. For example, the author could consider the Traits-Desires-Intentions-Behaviours Model (Miller 1994; 2011) which explores the difference between ‘desire’ and ‘intention’ in the context of reproductive decision-making. The authors ought to either use consistent terminology in this case and refer to the same term that was used on the survey to participants, or be really clear on different definitions if using multiple terms. Definitions should also be delineated for how the authors are interpreting ‘environmental concern’. Finally, acronyms need to be expanded when first used in the text which applies to line 210 and line 246.

**Response:** The term “childbearing aspirations” means aspiration or intention to have children. So, the use of terms such as: childbearing aspirations and environmental concern are interpreted according to the study. The authors use consistent terminology such as aspiration/ intention to have children. The definitions of the term have also been included. And acronyms have been expanded: Physics, Forestry and Environmental Science, Chemical Engineering & Polymer Science, Extreme Weather Events.

4. Methodology/Results: certain elements of the methodology and results sections require greater clarification. Examples are listed below:

There ought to be a reference for the ‘Research Randomizer’ tool.

**Response:** Reference has been added

Line 270-272 and 278-281: the authors state that only three out of eight questions met the necessary criteria. How does this validate the tools if less than 50% were satisfied?

**Response:** The confusion has been cleared.

Line 290: gender percentage is incorrectly reported in the text (correct to 48.2% aligned with Table 5).

**Response:** Corrected. You can see the changes in the results section.

Line 296: this is the only time Agricultural and Mineral Science is referred to as a participant’s degree. This puts the percentage for degrees off by +0.5%. The authors should clarify if this was a participant/s degree or not and adjust the text and table 5 accordingly.
The survey questions ought to be included in the appendix to give readers insight into the tools used.

**Response:** The confusion has been cleared. The Department of Forestry and Mineral Science, under the Faculty of Agricultural and Mineral Science, was included in the survey. However, as it is the only department within this faculty and only one student participated—due to the master's and fourth-year classes having ended and the semester exams having started during data collection. Therefore, we excluded this department from our analysis. Also, the survey question has been included.

5. Limitations: the limitations section could be expanded to include, for example, self-reporting bias inherent to surveys, and the issues with using binary measures for complex variables e.g., classifying homes as vulnerable or not vulnerable to climate change (line 242).

**Response:** Limitation section has been expanded according to the your suggestion.

6. Discussion: The discussion would benefit from further exploration of the broader range of factors influencing childbearing decisions. Whilst the study is focussed on the impact of environmental concern, other considerations – such as personal finances, partner preferences, social support etc. – are also likely to influence student’s opinions so it is worth addressing this, even if briefly in a couple of sentences.

**Response:** Added in discussion section

7. Conclusion: While the data supports the conclusions made, the framing that students who are environmentally concerns are more likely to want children, yet want fewer of them is confusing. It should be clarified that while participants with environmental concerns may still want children, the findings also showed that they plan on limiting family size due to these concerns. This framing should also be addressed in the abstract (line 24). Additionally, the conclusion should be limited to a concise summary of the study which avoids the introduction of new ideas.

**Response:** Conclusion and abstract section have been modified.

Reviewer #2: Introduction

Avoid overusing "laundry lists" when constructing paragraphs, as they can obscure the main message the author wants to convey. For example, in lines 75–79, it would be more effective to summarize the points about family planning in 1–2 concise sentences.

**Response:** Thank you for comment. The sentences have been removed as it is not well-fitted in the manuscript.

Line 81: The introduction of the Bangladesh context feels abrupt and disconnected in this paragraph. Consider providing context first—such as explaining why densely populated countries might be more impacted by future childbearing aspirations. Then, introduce Bangladesh as an example, along with other similarly populated countries, to offer a more comprehensive perspective.

**Response:** Thank you for your suggestion. You can see the changes: Introduction section has been split and a new heading has been added, “context of the study: Bangladesh

Line 96: “The question, “How do university students in Bangladesh, who are more aware of family planning and concerned about the environment, make their decisions about having children in the future?” lacks sufficient explanation and justification. It is unclear why university students are presumed to be more aware of family planning or have heightened environmental concerns. Providing data or references to support these assumptions would strengthen the argument and improve clarity.

**Response:** Thank you for your comment. The statement has been modified and provided reference as well.

Methods and Results

Line 234-235: repeated sentences.

**Response:** Corrected

Can you clarify whether you are using factor analysis or component analysis? Based on the wording, "Component analysis extraction technique" likely refers to principal component analysis (PCA). However, PCA is a data reduction technique and not ideal for establishing convergent validity compared to factor analysis.
 **Response:** Corrected. You can see the changes in the reliability and validity test.

Line 269: The phrase "average factor loading correlation" is unconventional. Factor loadings and correlations are distinct concepts.

**Response:** The confusion has been cleared.

Is there any specific reason to analyze the environmental concerns variable items independently? Please elaborate.

**Response:** For selecting the impact of environmental concern on future childbearing as an independent variable: each item was analyzed separately to capture the specific aspects of concern due to the multidimensional nature of environmental concern. In addition, this item did not show a high Cronbach's Alpha value, therefore, independent analysis enables nuanced understanding of each concern instead of using it as a combined score. This justification has also been included in the manuscript.

Discussion,

Line 492: How do the data of Americans and Chinese relate to the findings?
A more thorough review in the introduction will enable the authors to produce a more empirically and theoretically informed discussion.

**Response:** Corrected. You can see the changes in the discussion section.

Overall

Many variables are examined in the study; however, the lack of a comprehensive literature review limits the depth of the discussion on the findings. For instance, in lines 506–508, how can the increased willingness to have children among respondents with specific demographic characteristics be explained? For example, Muslim respondents are more likely to express a desire for future childbearing. Could this tendency be related to their religious beliefs?

**Response:** Thank you for your suggestion. Specific demographic variables were examined in the study, and these variables have been included in the literature review section. You can see the changes in “Factors associated with fertility decision making” and “Discussion” section.

Reviewer #3: Thank you for letting me read what seems to be a methodologically stringent and sound study. Before being publishable, I nonetheless think the manuscript needs to strengthen its relevance for both theory and practice. I have some suggestions for this at the end of my comments.
1. The very first paragraph is about environmental concerns in general, resulting in a bit vague introduction. If the topic is how such concerns are related to fertility intentions, then this (including why this topic is important) should be presented already in the first paragraph.
The description of the Bangladesh context is useful, and should perhaps have its own header? For instance, “Context of the study”.

**Response:** Thank you for your suggestion. You can see the changes in Introduction. A header “Context of the study Bangladesh” has been added.

2. I am not very experienced with quantitative methods, but I nonetheless did not catch a rationale for having dichotomous items for the variable “The impact of environmental concerns on future childbearing”. Beyond this, my comments are indeed restricted by the fact that I am not well trained to see limitations in the described procedures and statistical analyses.

**Response**: Thank you for raising this issue. We acknowledge that a Likert scale could have provided more nuanced insights into the degree of influence. We have also addressed this concern in the limitation section. However, we choose a dichotomous scale to capture a clear distinction between individuals who perceive environmental concerns as influencing their childbearing decisions and those who do not. This approach simplifies analysis and eases of interpretation in statistical modeling.

3. What I do think this manuscript is lacking, is a clearer idea of how and why the results are relevant. For instance, I do not think the statement “This study plays an important role in attaining the Sustainable Development Goals (SDGs) in Bangladesh” (p. 34-35) is justified by the manuscript. The relation to the SDGs has to be elaborated on, and I also think the manuscript should go in dialogue with the article “At the intersection of climate justice and reproductive justice” (Sasser, 2024, <https://doi.org/10.1002/wcc.860>).

**Response:** Thank you for suggestion. We remove the statement “This study plays an important role in attaining the Sustainable Development Goals (SDGs) in Bangladesh” because it is not well-fitted for the manuscript. However, we cited the Sasser (2024) work in the literature section and incorporate relevant discussions on climate justice and reproductive justice related to our findings, you can see in the discussion section.
