# Supplementary material for: The nexus between environmental concern and future childbearing aspirations among university students in Bangladesh
Source: PLoS One. 2025 Jun 4;20(6):e0325369. doi: 10.1371/journal.pone.0325369 (PMC12136327; doi:10.1371/journal.pone.0325369)
Supplement: Attachment — Submitted filename: Response to reviewers.docx [file pone.0325369.s004.docx]

**Editor Comments**

The structure also should be enhanced: there are separate analysis on which the rationale and the rationale of the sequence is not clear. Also regarding whether to use a scale vs all dichotomous items. The methods section and the research objectives should be made clearer so that the analysis flows from these. There is also a problem on sample size that might not be adequate for some of the more sophisticated analysis. As you know, due to multicollinearity, variances in multiple variable methods are higher than for simple two-variable analysis that you have used to ascertain sample size. This should be acknowledged as a limitation.

**Response:** Thank you for pointing this out and recommendations. We revised the structure of the ‘data analysis procedure’ section. You can see the changes. We acknowledge that a Likert scale could have provided more nuanced insights into the degree of influence. We have also addressed this concern in the limitation section. However, we choose a dichotomous scale to capture a clear distinction between individuals who perceive environmental concerns as influencing their childbearing decisions and those who do not. This approach simplifies analysis and eases interpretation in statistical modelling. In addition, this item did not show a high Cronbach’s Alpha value; therefore, analysing the items independently as dichotomous variables enables a nuanced understanding of each concern instead of using it as a combined score. This justification has also been included in the manuscript. Moreover, while our sample size is adequate for descriptive and bivariate analyses, it may be limited for more complex multivariate models. We acknowledge this as a limitation in the manuscript.

In addition, citation style is not according to PLOS ONE numbered style. This needs to be fixed.

**Response:** Thank you for your comment. We have corrected the citation style according to the PLOS ONE numbered style.

The questionnaire, currently included as an appendix to the main manuscript, should be provided as a separate file.

**Response:** Corrected

Regarding the first reviewer, who was unavailable, apparently the points have been addressed. The second reviewer provides some optional suggestions. Reviewer 3 indicates that language must be improved. The editor concurs on the need to copy-edit the manuscript.

Examples of issues that should be addressed, but there are many. The whole writing up needs to be improved.

- L. 83: The reading is now very different. “Highly productive” does not seem to apply given the location of Bangladesh in GDP rankings. The beautifulness of the rivers is also a subjective term such as those reviewer 1 was indicating in the previous revision.

**Response:** Thank you for the comment. The confusion has been cleared.

- L. 196-198: Remove consideration on the buildings of SUST unless it is deemed relevant for some reason. Discussion should be based on academic program and level.

**Response:** Sentences have been removed.

- L. 245: literature should be singular.

**Response:** Corrected

- Example of needed rephrasing is “measurement and analysis”. Some issues with language: “The researchers coded the following predictors into

**Response:** Measurement and analysis rephrased into ‘Data analysis procedure’. The statement regarding “The researchers coded the following predictors into” has been removed as it is not well-fitted in the manuscript.

- 269 categorical variables for binary logistic regression and other analyses:”. Why mixing the analysis with the coding? Was the coding ex-post carried out by the researchers as this sentence implies? It seems this was a closed questionnaire so that you should not mention “coding” at all. More important: we do not need to know the numbers of the codes at all! Also, on this: “which faculty 272 members are currently studying” you mean “field of study”. As commented, grammar needs to be revised and improved.

**Response:** The confusion has been cleared and modified according to your suggestion.

- L. 278: What is meant by “graphical distributions were created using RStudio”. You mean graphics were produced with R (RStudio is the gui. “Graphical distributions” is meaningless. Statistical distributions are not graphical.)

**Response:** Corrected.

- Figure 3: The axis should be clearly identified: percentage what?

**Response:** Corrected all of the figures.

- Table 5 should include the respective Ns as a new column.

**Response:** Corrected.

- Table 6 is missing N

**Response:** Corrected.

- L. 467 and later: It is not specified whether the poisson regression includes also those reporting not wanting to have children codified as 0 children. It should, otherwise you are not dealing with the complete distribution.

**Response:** The Poisson regression model was conducted only among respondents who reported intending to have children, as the follow-up question on number of desired children was asked conditionally (You can see the questionnaire and Table 4). As such, individuals who stated they did not intend to have children were not included in the model.

- For discussion: You are including as a variable “Vulnerability to home locality” (which probably should be “vulnerability of”. Note this is student’s perception. It might well be that students living in the same locality fill this up differently.

**Response:** Modified and rephrased into perceived vulnerability of home locality to climate change or extreme weather events.

Review Comments to the Author

Reviewer #2: Context of Study: While the paper provides an environmental description of Bangladesh, it would be beneficial to include a more detailed demographic context, particularly characteristics relevant to the studied participants (e.g., religious beliefs, education levels, socioeconomic status). Additionally, comparing these demographics to national data would help illustrate the representativeness of the sample and strengthen the study’s generalizability.

**Response:** Thank you for your comment. We have included national data to represent the sample. We have also compared the national data in our findings. You can see the changes in the ‘Context of the Study’ and ‘Result-Descriptive Statistics’ sections.

Limitations: Has any effort been made to ensure that respondents interpreted the questions consistently? While it is commendable that the study acknowledges its limitations, it would be helpful to outline any steps taken to minimize potential misunderstandings, such as piloting the survey, providing clarifications, or conducting follow-up checks. This would enhance the study’s methodological rigor.

**Response:** Thank you for your suggestion. To ensure the clarity and consistency of the questionnaire, a pilot test was conducted with 12 participants (you can see this data collection techniques section). You can also see the changes in the limitation section.

Reviewer #3: I appreciate the work you have put in to respond to all three reviewers, and I think your manuscript is a lot better now. My two main concerns now are language and the use of Sasser (2024).
- Language: I find parts of the manuscript to be a bit hard to read, as many sentences are long and sometimes confusing. You should go through the whole manuscript trying to make the language clearer. Example: "The analysis revealed that factors such as gender, religion, CGPA, disciplinary backgrounds, home locality susceptible to climate change or EWEs, childlessness have a beneficial effect on the environment, and going childless is a better way to help the environment than recycling, found to be statistically significant." (p. 30, lines 536-540). Furthermore, the last two factors listed here seem more like items to me?

**Response:** Thank you for the suggestion. The sentence has been modified. We have also improved the language with clarity.

- Sasser (2024): You write that you have tried to "incorporate relevant discussions on climate justice and reproductive justice related to our findings". The way I read your discussion, you refer briefly to Sasser (2024) without actually discussing how your findings relate to her arguments. This must be fixed.

**Response:** Modified. You can see the arguments in the discussion section.
