# Supplementary material for: The nexus between environmental concern and future childbearing aspirations among university students in Bangladesh
Source: PLoS One. 2025 Jun 4;20(6):e0325369. doi: 10.1371/journal.pone.0325369 (PMC12136327; doi:10.1371/journal.pone.0325369)
Supplement: Attachment — Submitted filename: Response to reviewers (3).docx [file pone.0325369.s005.docx]

**Editor Comment:**

In addition, there are still opinionated terms that should be avoided. One instance is the expression "adverse reproductive consequences" that appears twice. You should replace it by a non-opinionated term such as lower reproductive intention or something along this line. In general the complete sentence " Our research also indicates that environmental degradation 645 can result in population decline due to adverse reproductive consequences. " shoud be removed since this is clearly beyond the analysis of the paper.

**Response:** Thank you for your suggestion. We removed the statement from this draft.

**Journal Requirements:**

Please review your reference list to ensure that it is complete and correct. If you have cited papers that have been retracted, please include the rationale for doing so in the manuscript text, or remove these references and replace them with relevant current references. Any changes to the reference list should be mentioned in the rebuttal letter that accompanies your revised manuscript. If you need to cite a retracted article, indicate the article’s retracted status in the References list and also include a citation and full reference for the retraction notice.

**Response:** No retracted articles included in the manuscript.

Reviewer #3: I appreciate that you have elaborated on how your work might relate to Sasser's. It will probably seem to you that I have a hang-up on her work, but since I now have commented on this aspect of your manuscript, I think I am obliged to follow up on it. The way you discuss your results in relation to Sasser, is still not convincing to me. Frankly, I find it a bit hard to follow what your argument actually is, other than that you both address reproductive issues and environmental concerns. I read your discussion as if you are saying that it makes less sense to view reproductive issues as a product of individual concern, when the current context is one of environmental degradation (i.e. that the context of environmental degradation is more relevant than individuals' concerns when it comes to reproductive choices). If this is what you are saying, this should be spelled out more clearly. If you, on the other hand, argue that your engagement with Sasser is mostly through that you both emphasize climate anxieties, this should also be made clearer. If there is a combination, as suggested by the sentence "While the result reflects the environmental concern, it also raises critical questions about whether such beliefs are freely chosen or shaped by structural factors", then this must also be written out as clearer arguments based on your results and how they contribute to Sasser's framework.

**Response:** Thank you for your thoughtful follow-up comment. We appreciate your continued attention to how our paper relates to Sasser’s ideas. We now see that we could have explained more clearly how our findings connect with her argument. In the revised version of the paper, we explain it with clarity. We hope this more explicit engagement will address your concern and clarify our position in relation to Sasser’s work. You can see the changes in discussion section.

My specific concern with how you discuss Sasser, mirrors a more general concern regarding the clarity of your discussion. For instance, when you have revised the sentences on bottom p. 31 and top p. 32, I think you could still do more to make the language clear, correct, and unambiguous. To illustrate what I mean, I'll provide specific suggestions for these two sentences:

Current: "The analysis revealed that factors such as gender, religion, CGPA, disciplinary backgrounds, perceived vulnerability of home locality to climate change or EWEs were significantly associated with future childbearing intention."
Suggestion: Add an "and" before "perceived".

**Response:** Thank you for your comment. You can see the changes

Current: "In addition, two specific items- childlessness has a beneficial effect on the environment, and going childless is a better way to help the environment than recycling- were also found to be statistically significant."
Suggestion: "In addition, two specific items (i.e. "childlessness has a beneficial effect on the environment"; "going childless is a better way to help the environment than recycling") were also found to be statistically significant."

**Response:** Corrected.
