# Supplementary material for: The nexus between environmental concern and future childbearing aspirations among university students in Bangladesh
Source: PLoS One. 2025 Jun 4;20(6):e0325369. doi: 10.1371/journal.pone.0325369 (PMC12136327; doi:10.1371/journal.pone.0325369)
Supplement: Attachment — Submitted filename: Response_to_reviewers_auresp_4.docx [file pone.0325369.s006.docx]

**Editor Comment:**

**My specific comments on avoiding opinionated terms has been implemented, but I am missing among the limitations of the study an acknowledgement that this is an observational study and that no causal relation between specific concerns and childbearing intentions can be inferred. There could be non-causal mechanisms such as people with certain traits having correlated opinions and attitudes in the dimensions explored. Please introduce a paragraph along these lines in the limitations, and the paper could be published.**

**Response:** Thank you for your suggestion. We included your suggested lines in the limitations section. We hope that you will find our revised version publishable now.
